# Adaptive Selection of Truncation Radius in Calderon’s Method for Direct Image Reconstruction in Electrical Capacitance Tomography †

**DOI:** 10.3390/s19092014

**Published:** 2019-04-29

**Authors:** Shijie Sun, Lijun Xu, Zhang Cao, Jiangtao Sun, Wenbin Tian

**Affiliations:** 1 School of Instrumentation and Optoelectronic Engineering, Beihang University, Beijing 100191, China; sunsj@buaa.edu.cn (S.S.); zh_cao@buaa.edu.cn (Z.C.); jiangtao_sun@buaa.edu.cn (J.S.); wenbin_tian@buaa.edu.cn (W.T.); 2 Beijing Advanced Innovation Center for Big Data-Based Precision Medicine, Beihang University, Beijing 100191, China; 3 School of Computer Science and Engineering, Beihang University, Beijing 100191, China

**Keywords:** electrical capacitance tomography, industrial process tomography, direct image reconstruction, Calderon’s method, truncation radius

## Abstract

Calderon’s method has been successfully used for the direct image reconstruction in electrical capacitance tomography. In the method, the truncation radius adopted in numerical integral greatly influences the reconstruction results. In the past, the truncation radius is selected as a constant empirically according to the permittivity distribution pattern and noise level. In this paper, the influence of the truncation radius in Calderon’s method on the reconstruction results was first analyzed by numerical simulation. Then, a strategy for adaptive selection of the truncation radius was proposed. The amplitude information of the elements in the scattering transform matrix computed from the Dirichlet-to-Neumann (DN) map was used to determine the range for the truncation radius selection, and the phase information was further used to select a proper truncation radius value within this selection range. Finally, experiments were carried out to verify the strategy. Experimental results showed that small relative image error and good visual effect could be obtained by using the truncation radius selected by the proposed strategy.

## 1. Introduction

Electrical capacitance tomography (ECT) is an imaging technique to obtain the permittivity distribution in the region of interest from capacitance measurements on the external boundary [1]. The ECT technique has been developed since the 1980s and widely used for process monitoring in industrial applications, e.g., two-phase flow [2,3,4], fluidized beds [5] and flame monitoring [6,7]. Compared with other process tomographic techniques based on ultrasound, optics, X-ray and gamma-ray, it has several advantages including low cost, rapid response, good portability and radiation-free [8]. A typical ECT system usually consists of three main parts [9]: (1) An ECT sensor, (2) a data acquisition and processing unit, and (3) a computer for image reconstruction.

With the ECT data acquisition system, the capacitances between every two electrodes of the ECT sensor can be measured [10]. Then the permittivity distribution can be reconstructed by using a suitable reconstruction method. Because of the ‘soft-field’ property, the image reconstruction in ECT, i.e., recovering permittivity distribution from surface capacitance measurements, is a non-linear inverse problem and is severely ill-posed [11]. Therefore, the performance of image reconstruction method is crucial to ECT.

In the literature, most image reconstruction methods are implemented based on the sensitivity theorem [12], e.g., the one-step methods including linear back projection (LBP) method [13], Tikhonov method, singular value decomposition (SVD) method [14] etc., and the iterative methods including the Landweber method [15], algebraic reconstruction technique (ART) method [16], etc. These methods have been widely used in many applications. However, the intrinsic ‘soft-filed’ property of ECT in the sensing region is ignored in these cases [17].

Besides the sensitivity-based methods, another one-step method for direct image reconstruction is introduced by Calderon [18] and has been successfully applied in ECT [17,19,20]. Unlike the sensitivity-based methods, the boundary map matrix is used in the Calderon’s method and the two-dimensional Fourier transform is applied to reconstruct the perturbations of the permittivity distribution. Calderon’s method can provide the gray value at any pixel of the reconstructed image using a direct and independent approach. It is time-saving as no matrix inversion or iterative process is involved [19].

Several parameters need to be determined when using the Calderon’s method, i.e., the number of electrodes, integral nodes and truncation radius used in the numerical integral. (1) If more electrodes are used in ECT, the quality of the reconstructed image can be improved, but the hardware burden will increase. The number of electrodes is generally selected as 12 or 16. (2) More integral nodes will increase the accuracy of the integral process but result in a long computing time. In our previous study, the number of integral nodes was selected as 30 [21]. (3) Increasing the truncation radius will increase the high-frequency components in the reconstructed image, but also increase the image noise. In practical applications, the truncation radius is usually set as a constant empirically at present. However, when we apply the Calderon’s method to the monitoring of industrial processes, e.g., two-phase flows [22], the selection of initial truncation radius greatly influences the reconstructed results and the recognition of flow patterns. For example, it is appropriate to choose a small truncation radius for laminar flows and a large truncation radius for core-type flows. Since the true distribution is usually unknown, it is difficult to select a suitable truncation radius. Therefore, it is necessary to find a method to adaptively select the truncation radius with respect to different permittivity distributions.

In this paper, the basic fundamental of the Calderon’s method is first presented. Then numerical simulations are carried out to analyze the influences of the truncation radius in the Calderon’s method on the reconstructed results with four typical permittivity distributions. A method is proposed to adaptively select the truncation radius using the amplitude and phase information of the scattering transform, which can improve the applicability of the Calderon’s method.

## 2. Calderon’s Method for Direct Image Reconstruction in ECT

**Figure 1:**
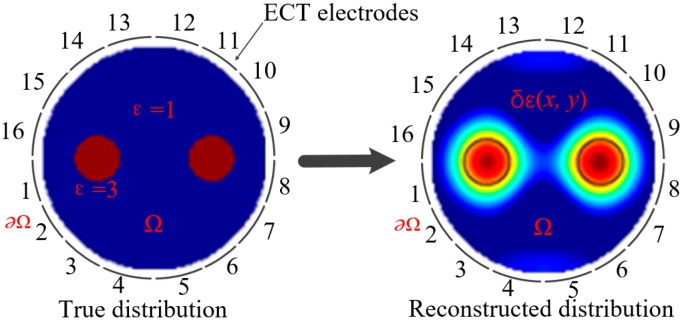
A typical electrical capacitance tomography (ECT) sensor.

For ECT, the governing equation of the sensing field, denoted by Ω, is:

(1) ∇⋅ε(z)∇φ(z)=0

where *z* represents the point with coordinates (*x, y*). *ε*(*z*) and *φ*(*z*) are the permittivity and electrical potential at *z*. Figure 1 shows a typical ECT sensor.

According to the divergence theorem, the relationship between the integrals of the spatially varying permittivity and voltage and current measurements on the boundary is:

(2) $$\int_{\Omega }\upsilon (z)\nabla \cdot \varepsilon (z)\nabla \varphi (z)dz=\int_{\partial \Omega }\upsilon (z)\varepsilon (z)\frac{\partial \varphi (z)}{\partial n}dl-\int_{\Omega }\nabla \upsilon (z)\cdot \varepsilon (z)\nabla \varphi (z)dz=0$$

where *υ(z)* is an arbitrary continuous function in L2-space and *dl* is the measured arc length on the boundary.

The Dirichlet-to-Neumann (DN) map takes the given voltage distribution on the boundary to the current density distribution, which is also called the voltage-to-current-density map and can be expressed as:

(3) $$\Lambda_{\varepsilon }:\varphi (z){|}_{\partial \Omega }\to \varepsilon (z)\frac{\partial \varphi (z)}{\partial n}{|}_{\partial \Omega }$$

where $\Lambda_{\varepsilon }$ denotes the DN map when Ω contains *ε*(*z*). Equation (2) can be rewritten as:

(4) $$\int_{\Omega }\varepsilon (z)\nabla \upsilon (z)\cdot \nabla \varphi (z)dz=\int_{\partial \Omega }\upsilon (z)\Lambda_{\varepsilon }[\varphi (z)]dl$$

For a disturbed permittivity *ε*(*z*) = 1 + *δε*(*z*), if this change only exists in Ω and $\varphi_{1+\delta \varepsilon }(z)\approx \varphi_{1}(z)$, we obtain:

(5) $$\int_{\Omega }\delta \varepsilon (z)\nabla \upsilon (z)\cdot \nabla \varphi_{1}(z)dz\approx \int_{\partial \Omega }\upsilon (z)(\Lambda_{1+\delta \varepsilon }-\Lambda_{1})[\varphi_{1}(z)]dl$$

In Calderon’s method, we construct that $\varphi_{1}(z)=e^{ikz}$ and $\upsilon (z)=e^{i\overset{-}{kz}}$. The right side of Equation (5) is defined as the scattering transform, *t*(*k*), and *k* = *k*_1_ + *ik*_2_. Then Equation (5) becomes:

(6) $$t(k)\approx \int_{\Omega }\delta \varepsilon (z)\nabla \upsilon (z)\cdot \nabla \varphi_{1}(z)dz=-2(k_{1}^{2}+k_{2}^{2})\iint_{\Omega }\delta \varepsilon (x,y)e^{-i(-2k_{1},2k_{2})\cdot (x,y)}dxdy$$

here, the scattering transform can be regarded as the two-dimensional Fourier transform of the permittivity distribution.

Then the permittivity change can be obtained by using inverse Fourier transform,

(7) $$\delta \varepsilon (x,y)\approx \frac{1}{2\pi^{2}}\iint_{R^{2}}\frac{t(k_{1}+ik_{2})}{k_{1}^{2}+k_{2}^{2}}e^{i(-2k_{1},2k_{2})\cdot (x,y)}dk_{1}dk_{2}$$

In the polar coordinates of parameters *r* and *θ*, Equation (7) can be rewritten as:

(8) $$\delta \varepsilon (x,y)\approx \frac{1}{2\pi^{2}}\int_{0}^{R}\int_{-\pi }^{\pi }\frac{t(re^{i\theta })}{r}e^{i2r(-cos\theta ,sin\theta )\cdot (x,y)}d\theta dr$$

where *R* is the truncation radius of the region used in the numerical integral.

The scattering transform has been approximated and linearized to provide practical and simpler implementations in the D-bar method [23]. Since Calderon’s method is an approximation of the D-bar method, the approximation method of scattering transform *t*(*k*) is also used here. When we consider the noise in practical measurements, *t*(*k*) needs to be truncated, which can be expressed by:

(9) $$t(k)\approx \{\begin{array}{ll} \int_{\partial \Omega }e^{i\overset{-}{kz}}(\Lambda_{1+\delta \varepsilon }-\Lambda_{1})e^{ikz}dl, & \left|k\right|\leq R\\ 0, & \left|k\right|>R \end{array}$$

The truncation radius, *R*, is the upper limit of the frequencies in the inverse Fourier transform process.

## 3. Adaptive Selection of Truncation Radius

### 3.1. Influence of the Truncation Radius on Reconstructed Results in Spatial Domain

**Figure 2:**
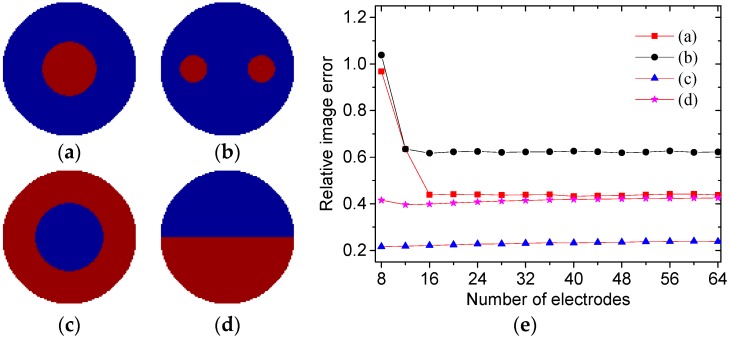
Four typical permittivity distributions: (**a**) Core, (**b**) multiple objects, (**c**) annular and (**d**) stratified. (**e**) Relative image errors against number of electrodes for the four distributions.

In Calderon’s method, three parameters need to be pre-determined, i.e., the number of electrodes, integral nodes and truncation radius used in the numerical integral. As the integral nodes have been studied and selected as 30 in our previous work, the number of electrodes and truncation radius were analyzed here. In the following, four typical permittivity distributions, i.e., core, multiple objects, annular and stratified, were simulated, as shown in Figure 2a–d. The red color represents the region with high permittivity (*ε* = 3) and the blue color represents the region with low permittivity (*ε* = 1). The finite element method (FEM) was used to solve the forward problem, i.e., to obtain the boundary capacitance values from different permittivity distributions.

From Equation (4), increasing the number of electrodes in ECT can improve the computational accuracy of the scattering transform. However, increasing the number of electrodes will increase the hardware burden. To analyze the influences of the number of electrodes on the reconstructed results, the variations in the relative image errors against the number of electrodes for the typical permittivity distributions are shown in Figure 2e. The relative image error between the true permittivity distribution and reconstructed image can be expressed by:

(10) $$err=\frac{\left\|g_{r}-g\right\|}{\left\|g\right\|}$$

where *err* is the relative image error, *g_r_* is the true permittivity distribution and *g* is the reconstructed permittivity distribution. The truncation radii are selected as 6, 5, 2.4 and 2.4 for the four permittivity distributions, respectively.

Figure 2e shows that the relative image errors decrease significantly if the number of electrodes changes from 8 to 16. When the number of electrodes is larger than 16, the relative image errors only change a little bit. With consideration of the hardware burden and image quality, the number of electrodes is selected as 16 in this paper.

**Figure 3:**
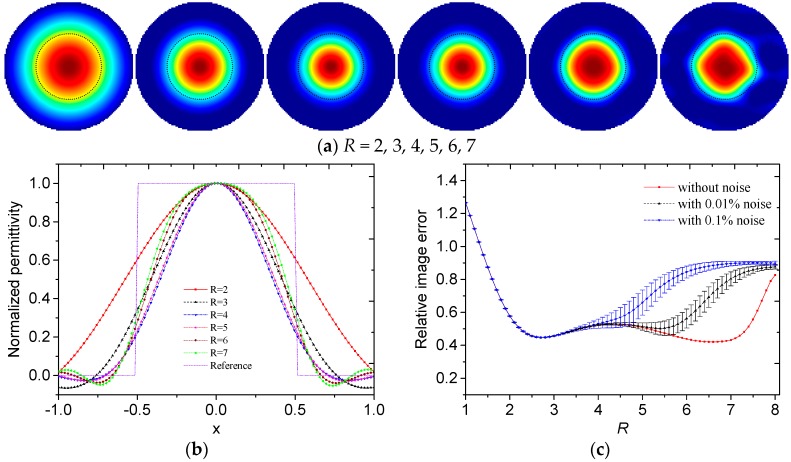
(**a**) Reconstructed images, (**b**) reconstructed radially symmetric permittivity distribution along the *x*-axis and (**c**) relative image errors with different truncation radii for core-type distribution.

**Figure 4:**
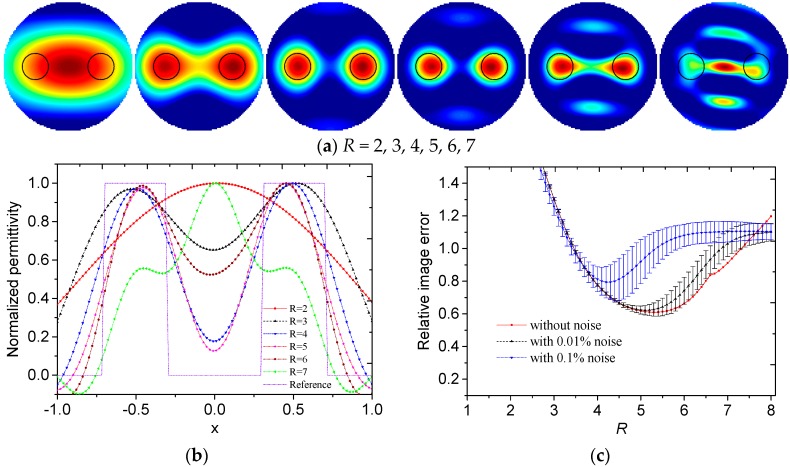
(**a**) Reconstructed images, (**b**) reconstructed radially symmetric permittivity distribution along the *x*-axis and (**c**) relative image errors with different truncation radii for multiple-object-type distribution.

**Figure 5:**
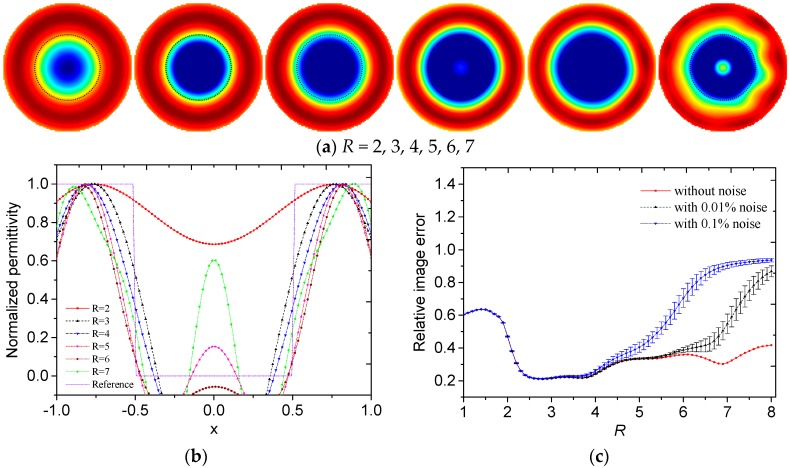
(**a**) Reconstructed images, (**b**) reconstructed radially symmetric permittivity distribution along the *x*-axis and (**c**) relative image errors with different truncation radii for annular distribution.

**Figure 6:**
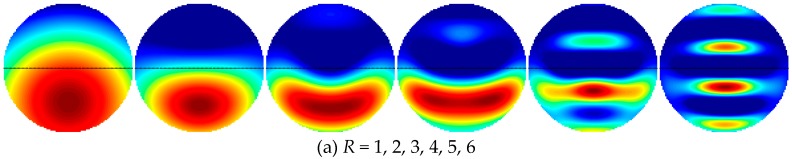

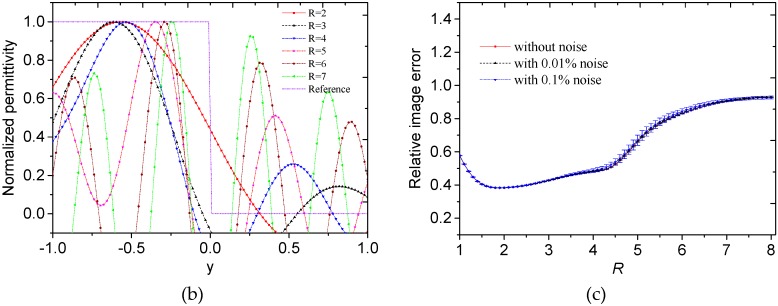
(**a**) Reconstructed images, (**b**) reconstructed radially symmetric permittivity distribution along the *y*-axis and (**c**) relative image errors with different truncation radii for stratified distribution.

Then the influences of the truncation radius on the reconstructed results are investigated. The reconstructed images of different permittivity distributions with different truncation radii and without noise are shown in Figure 3a, Figure 4a, Figure 5a and Figure 6a. The black dotted lines represent the edges of the original region with high permittivity. The reconstructed radially symmetric permittivity distributions along the *x*- or *y*-axis are shown in Figure 3b, Figure 4b, Figure 5b and Figure 6b. The reconstructed permittivity is normalized by dividing the maximum permittivity and setting the negative permittivity to 0. The negative permittivities are retained in these figures to show the influence of *R*.

The relationship between the relative image error and the truncation radius with different permittivity distributions is shown in Figure 3c, Figure 4c, Figure 5c and Figure 6c. The simulated capacitance values are contaminated with noise of relative magnitude 0.01% and 0.1%. The error bars are plotted using the standard deviations, which are calculated by repeating the reconstruction with noise-contaminated capacitance values for 100 times.

In Figure 3a and Figure 4a, a small *R* means that the reconstructed image contains few high frequency components. In the reconstructed images, the low frequency components contribute to the slowly-changing part, which represents the general overview and contour of the image. A large *R* means that the reconstructed image contains more high frequency components. More high frequency components can reconstruct sharp changes between adjacent image areas, such as edges and details in the image. However, more high frequency components also give rise to more noise. Therefore, it is important to select an appropriate *R* to make a trade-off in the Calderon’s method.

In Figure 3b, with *R* increasing, the slope of the normalized permittivity curve increased and the fluctuation of permittivity on the edge increased. In Figure 4b, with *R* increasing, the centers of the reconstructed circles became closer to the position of *x* = 0. When *R* = 4, the locations of the reconstructed circles were more accurate. By visual inspection, the suitable *R* was between 4 to 5 for core-type and multiple-object-type distributions.

In Figure 3c and Figure 4c, the truncation radius greatly influenced the relative image error. The truncation radii obtained using the minimum errors without noise for core-type and multiple-object-type distributions were 6.6 and 5.3. When the noise level increased, *R* with the minimum error decreased.

With consideration of the visual effect of the reconstructed image and the relative image errors, the truncation radius should be selected within a range between 4 and 6 for core-type and multiple-object-type distributions.

Figure 5 and Figure 6 show the reconstructed results of annular and stratified permittivity distributions. Unlike the core-type and multiple-object-type permittivity distributions, the region of high permittivity was on the edge of the sensing region. With consideration of the visual effect of the reconstructed image and the relative image errors, the truncation radius should be selected between 2 and 4, which were lower than that for core-type and multiple-object-type distributions.

The above analysis shows that the truncation radius cannot be set as a constant for ECT in various industrial applications. It is necessary to find a method to adaptively select a suitable *R*.

### 3.2. Influence of the Truncation Radius on Scattering Transform in the Frequency Domain

In this section, *R* was analyzed in the frequency domain. Equation (9) provides an approximation of *t*(*k*) in a grid inside the disc |*k*| < *R*. *t*(*k*) needs to be truncated by selecting a suitable *R*, which acts as a low-pass filter. Since the image reconstruction process, i.e., the calculation of δε(x,y), can be regarded as the two-dimensional inverse Fourier transform of:

(11) $$F(r,\theta )=\frac{t(re^{i\theta })}{2r}$$

the truncation radius, *R*, plays a role of limiting the frequencies in the inverse Fourier transform process. Increasing *R* means that the reconstructed images contain more high frequency components and the contours and boundary details of the objects become clearer. However, the noises and artifacts increase with *R* increasing.

**Figure 7:**
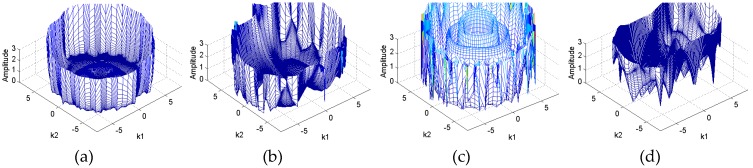
Amplitude distributions of *F* for different permittivity distributions: (**a**) Core, (**b**) multiple objects, (**c**) annular and (**d**) stratified.

Let us consider the spectral information of the reconstructed image, i.e., amplitude distribution and phase distribution of *F*. The amplitude distributions of *F* for different permittivity distributions are shown in Figure 7.

**Figure 8:**
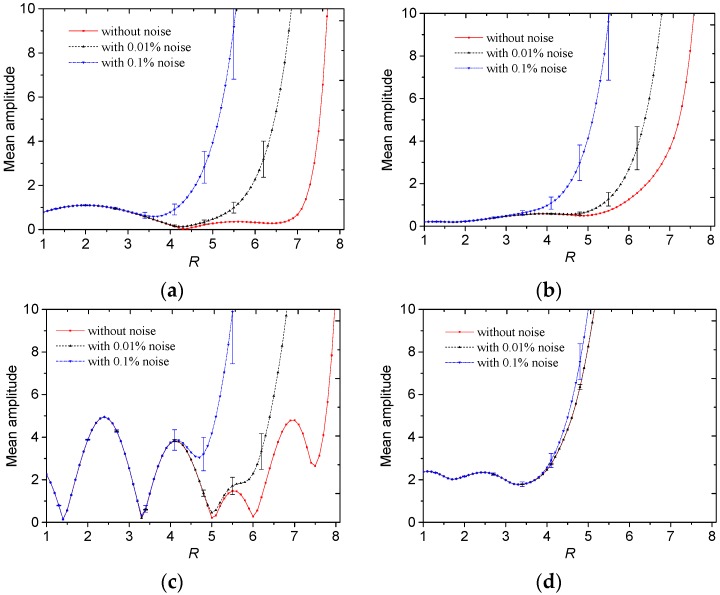
Mean amplitude of *F* against the truncation radius for different permittivity distributions: (**a**) Core, (**b**) multiple objects, (**c**) annular and (**d**) stratified.

To clearly show the variation tendency of the amplitude of *F* in Figure 7, the relationship between the truncation radius and the mean amplitude of *F* is shown in Figure 8. The mean amplitude of *F* is calculated by:

(12) $$\bar{F_{a}}(r)=\frac{\sum_{\theta =0}^{2\pi }mag(F(r,\theta ))}{N}$$

where *mag*() is the amplitude value of a complex number and *N* is the number of *θ.*

In Figure 8, the amplitude values without noise increased by a large scale from a fixed value of $\bar{F_{a}}(r)$, e.g., 5. If the noise level increases, the corresponding truncation radius decreases when $\bar{F_{a}}(r)$ = 5. The values of *F* located in the neighborhood of *R *= 0 were less affected by the noise. Then the maximum *R* could be determined by selecting a fixed mean amplitude value of *F*. For example, if this mean amplitude value was 5, the maximum truncation radii for the four distributions were lower than 8.

**Figure 9:**
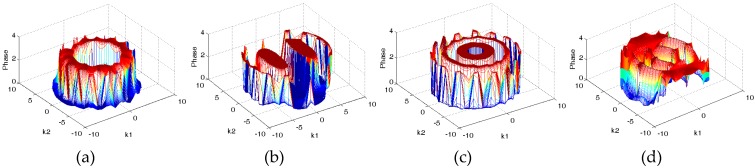
Phase distributions of *F* for different permittivity distributions: (**a**) Core, (**b**) multiple objects, (**c**) annular and (**d**) stratified.

Then we considered the absolute phase distributions of *F* for different permittivity distributions, which are shown in Figure 9.

**Figure 10:**
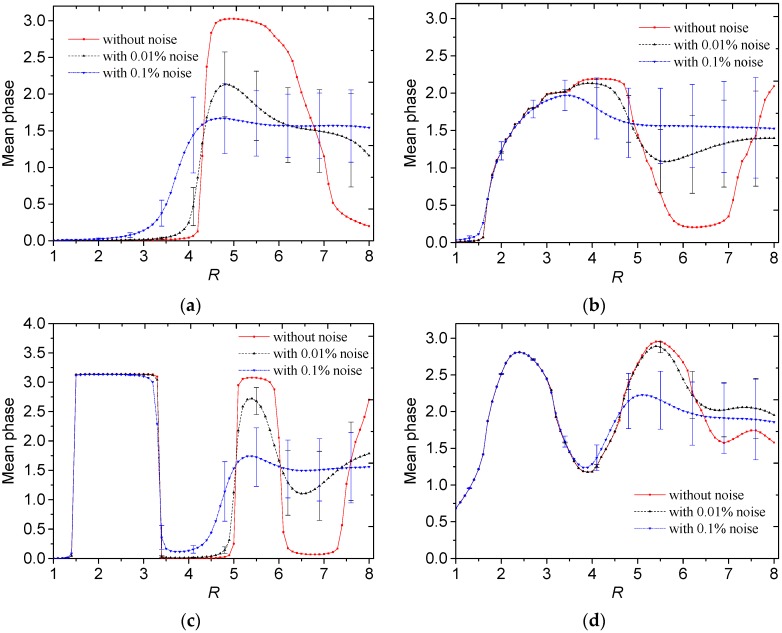
Absolute phase value of *F* against the truncation radius for different permittivity distributions: (**a**) Core, (**b**) multiple objects, (**c**) annular and (**d**) stratified.

Then the mean phase of *F* is calculated by:

(13) $$\bar{F_{p}}(r)=\frac{\sum_{\theta =0}^{2\pi }\left|angle(F(r,\theta ))\right|}{N}$$

where *angle*() is the phase value of a complex number. Then the relationship between the truncation radius and mean phase value of *F* is shown in Figure 10.

In Figure 10a,b, for the core-type and multiple-object-type distributions without noise, there existed one wave crest in the phase curve, and the peak value of phase located in the interval between *R* = 4 to *R* = 6. If the noise level becomes larger, the truncation radius becomes lower to reach the peak value. For the annular and stratified distributions in Figure 10c,d, there existed two wave crests in the phase curve, and the two peak values of phase locate in the intervals between *R* = 2 to 4 and 4 to 6. If the noise level becomes larger, the second wave crest is not obvious but the first wave crest still exists. From the reconstructed results in Section 3, the peak value of the absolute phase curve could be used as an index to select a suitable truncation radius.

### 3.3. Adaptive Selection of the Truncation Radius

*R* can be theoretically selected as [24]:

(14) $$R=-\frac{1}{10}\log\delta$$

where *δ* is the noise level. However, the suitable *R* for practical applications is generally much larger than the theoretical choice. In electrical impedance tomography for medical applications, *R* is usually selected between 4 and 6 empirically. Regarding ECT for industrial applications, the permittivity distribution changes dramatically. As described in the previous section, the choice of *R* in medical EIT is not suitable for annular and stratified distributions any more.

**Figure 11:**
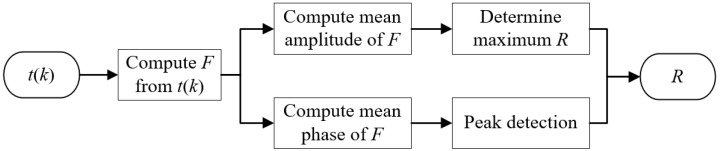
Flowchart of the strategy for adaptive selection of truncation radius.

From the above analysis, the amplitude and phase information of *F* can be combined to determine a suitable truncation radius. Firstly, the scattering transform *t*(*k*) is computed from the DN map. Then the truncation radius is selected using the following strategy for adaptive selection of *R*, as shown in Figure 11. When *t*(*k*) is obtained, the maximum *R* can be determined by a fixed mean amplitude of *F* and the minimum *R* can be set as a constant, which was selected as 2 in this paper. In this selection range of *R*, the peak value of $\bar{F_{p}}(r)$ could be detected and used to select the corresponding *R*.

**Figure 12:**
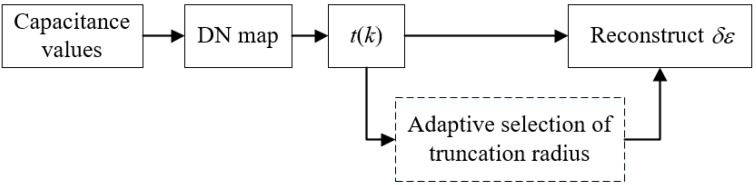
Schematic diagram of Calderon’s method with adaptively selected truncation radius.

The schematic diagram of Calderon’s method with adaptively selected truncation radius is shown in Figure 12. The capacitance values were measured by the ECT data acquisition system and used to construct the DN map. Then *t*(*k*) was computed from the DN map and *R* was selected by the proposed strategy. Finally, the permittivity change could be obtained by using Equation (8) with the selected *R*.

*R* selected by the proposed method and the corresponding relative errors for the four permittivity distributions are shown in Table 1. The minimum relative errors were obtained using *R* between 2 to 8 with a step of 0.1 for the four permittivity distributions, and the corresponding *R* are also shown in Table 1 for comparison. When the simulated capacitance values were contaminated with noises, the truncation radii and relative errors were the mean values from 100 times calculations.

Comparing Figure 3 and Figure 4 and Table 1, the truncation radii selected by the proposed method were in the suitable range between 4 to 6 for the core-type and multiple-object-type distributions, although they brought relatively higher image errors than the actual minimum errors. The truncation radii selected by the proposed method were lower than those with the minimum errors. However, with the selected *R*, fewer artifacts existed in the reconstructed images and the locations of the reconstructed circles were more accurate, which are beneficial for flow pattern recognition. When the noise level increased, the suitable truncation radius decreased.

For the annular and stratified distributions, the truncation radii selected by the proposed method were very close to those obtained according to the minimum errors, which were also in the suitable range between 2 to 4. The noise level had less impact on the selection of truncation radius for the annular and stratified distributions.

## 4. Experimental Results

**Figure 13:**
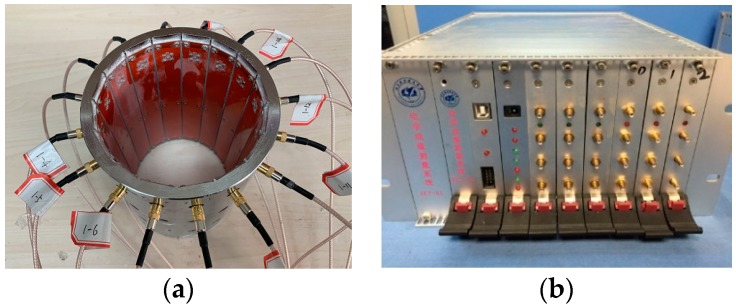
(**a**) ECT sensor and (**b**) ECT data acquisition system.

In this section, experiments were carried out to evaluate the performance of the proposed method. The number of electrodes in the ECT sensor was selected as 16. The inner diameter of the sensor was 100 mm and the length of the electrode was 120 mm, as shown in Figure 13a. The ECT system developed by Beihang University was used to measure the capacitance values between every two electrodes of the ECT sensor, as shown in Figure 13b. The signal-to-noise ratio (SNR) of the capacitance measurement with the ECT system was larger than 50 dB for the 16-electrodes sensor.

Similar to the simulation process, several typical permittivity distributions were constructed. Cylindrical nylon rods were used to construct the core-type and multiple-object-type distributions, i.e., D1 to D3. Several 3D-printed models and a certain amount of fine sand were used to construct the square-shaped, V-shaped, stratified and annular distributions, i.e., D4 to D9. The permittivities of the plastic rods and sand were about 3.

**Figure 14:**
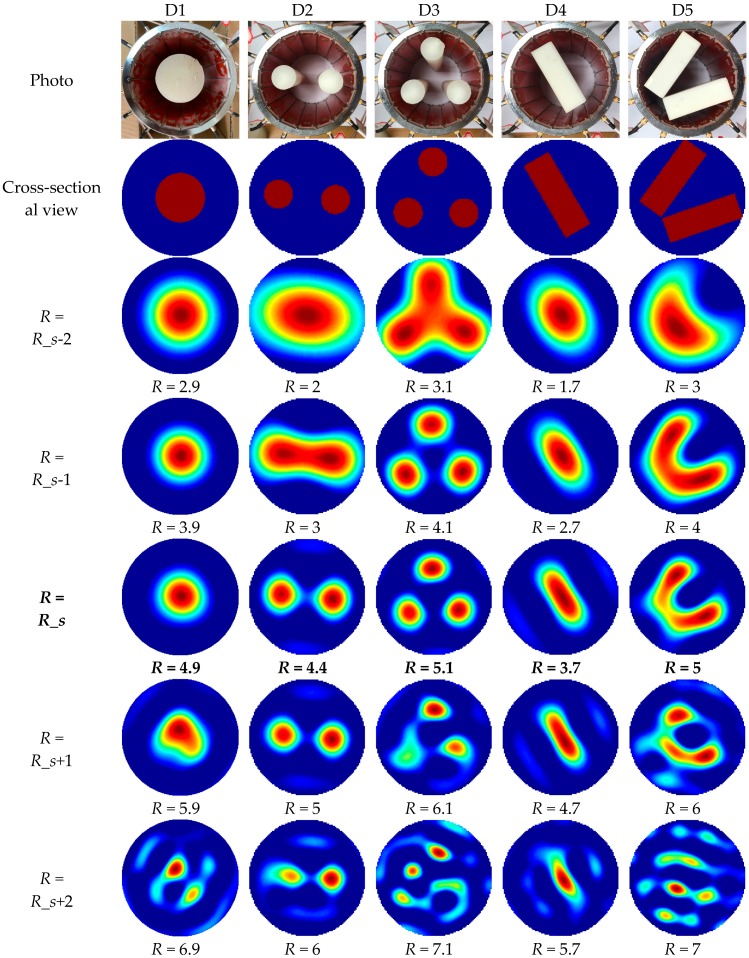
Photos, cross-sectional views and reconstructed images of different permittivity distributions from D1 to D5 with different truncation radii.

The permittivity distributions were reconstructed using the measured capacitance values and Calderon’s method with the proposed strategy for truncation radius selection. The truncation radius selected by the proposed method was denoted as *R_s*. The photos, cross-sectional views and reconstructed images of the core-type and multiple-object-type distributions are shown in Figure 14.

The selected truncation radii were 4.9, 4.4, 5.1, 3.7 and 5 for the distributions D1 to D5. From the visual point of view, when *R* was smaller than *R_s*, the reconstructed high-permittivity regions were excessively large. The low frequency components provided more slow-changing parts. When *R* was larger than *R_s*, more artifacts appeared, which meant that the high-frequency noise was introduced with *R* increasing. The high frequency components brought sharp changes between adjacent image areas.

**Figure 15:**
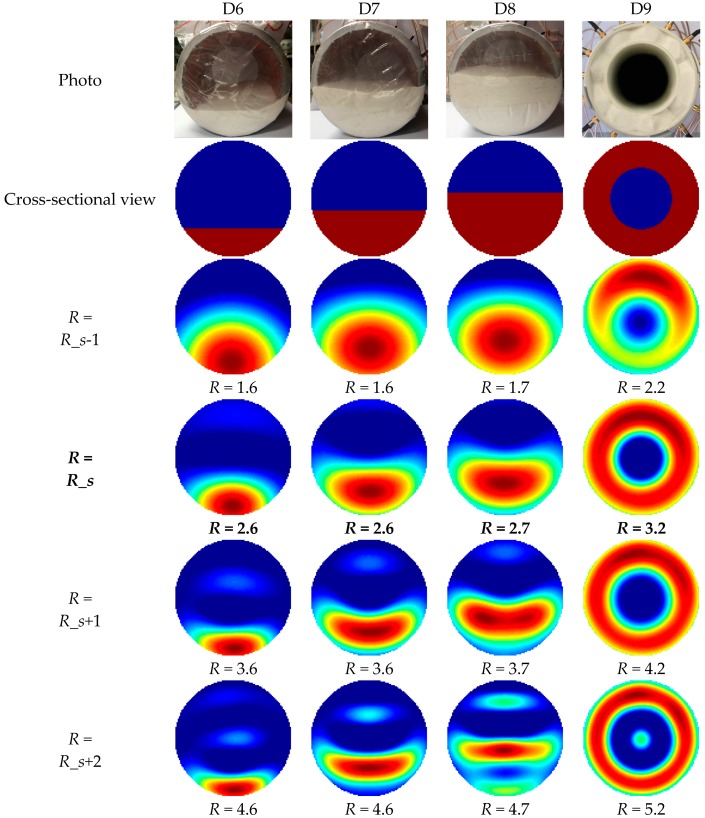
Photos, cross-sectional views and reconstructed images of different permittivity distributions from D6 to D9 with different truncation radii.

The photos, cross-sectional views and reconstructed images of the stratified and annular distributions are shown in Figure 15.

For the stratified and annular distributions D6 to D9, the selected truncation radii were 2.6, 2.6, 2.7 and 3.2, which were much smaller than those selected for distributions D1 to D5. By visual inspection, the selected truncation radii were also suitable for flow pattern recognition. It should be noted that the reconstructed images were visually good as well when *R* was a little larger than *R_s*, e.g., *R_s* + 1.

**Figure 16:**
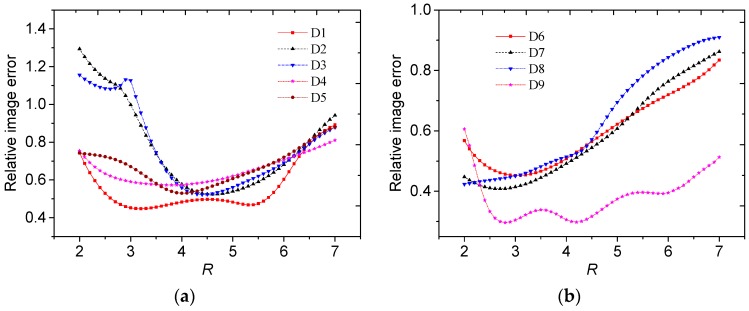
Relative image errors against the truncation radius for different permittivity distributions from (**a**) D1 to D5 and (**b**) D6 to D9.

To evaluate the reconstructed results with the selected truncation radius quantitatively, the relationship between the relative image errors and the truncation radius are shown in Figure 16.

From Figure 16, the relative errors using the selected *R* could be obtained, as shown in Table 2. Then we could obtain the minimum relative image errors for the permittivity distributions D1 to D9, and the corresponding truncation radii, which are also shown in Table 2 for comparison.

For all the distributions except for D3 and D5 in Table 2, the differences between the relative errors using the proposed method and the minimum relative errors were lower than 3%. For the distributions D3 and D5, the images reconstructed by the proposed method could reflect better contours of the objects, compared to the images with the minimum relative errors. There existed some contradictions between the visual effect of the reconstructed image and the relative image errors. The reason may be that there existed geometrical errors in the true distributions used for error calculation. With consideration of a tradeoff between the relative image error and visual effect, the truncation radii selected by the proposed method were in proper selection range.

## 5. Conclusions

A strategy for adaptive selection of the truncation radius was proposed by using the amplitude and phase information of the scattering transform. The following conclusions were drawn.

(1) A small truncation radius means that the reconstructed image contains few high frequency components. The low frequency components contribute to the slowly-changing part in the image. If the truncation radius becomes larger, the images contain more high frequency components and the contours and boundary details of the objects become clearer. However, the noises and artifacts increase;
(2) The permittivity distributions can be divided into two categories, i.e., the core-type and the annular distributions. For the core-type permittivity distributions, i.e., the region of high permittivity is enclosed by a continuous region of low permittivity, the truncation radius should be between 4 and 6 to obtain a better image quality. For annular and stratified distributions, i.e., the region of high permittivity contacts the boundary of the sensing region, the suitable truncation radius should be between 2 and 4, which is much lower than that for core-type distributions. For a higher noise level, a smaller truncation radius should be selected;
(3) The amplitude and phase information of the scattering transform are combined to determine a suitable truncation radius. The experimental results show that small relative image error and good visual effect can be obtained by using the truncation radius selected by the proposed method.

The main contribution of this paper was that a suitable truncation radius in Calderon’s method for direct image reconstruction could be adaptively obtained by using the information of scattering transform, without the need of the true distribution pattern. The scattering transform can be directly computed from the measured capacitance values, which makes the proposed method easy to implement and useful in industrial applications. 

## Figures and Tables

**Table 1:** Comparison of the relative errors and truncation radii obtained by the proposed method with those obtained according to the minimum relative error.

		*R* Selected by the Proposed Method	Relative Error Using the Selected *R*	*R* with Minimum Relative Error	Minimum Relative Error
Core	without noise	5	50%	6.6	42%
	0.01% noise	4.9	52%	3.1	45%
	0.1% noise	4.7	59%	2.8	45%
Multiple objects	without noise	4.1	75%	5.3	61%
	0.01% noise	4.1	80%	5.2	62%
	0.1% noise	3.8	96%	4.3	78%
Annular	without noise	2.4	23%	2.8	21%
	0.01% noise	2.4	23%	2.8	21%
	0.1% noise	2.2	30%	2.7	22%
Stratified	without noise	2.4	39%	1.9	38%
	0.01% noise	2.4	39%	1.9	38%
	0.1% noise	2.4	39%	1.9	38%

**Table 2:** Comparison of the relative errors and truncation radii obtained by the proposed method with those obtained according to the minimum relative error.

	*R* Selected by the Proposed Method	Relative Errors Using the Selected *R*	*R* with Minimum Relative Errors	Minimum Relative Errors
D1	4.9	48%	3.2	45%
D2	4.4	53%	4.6	52%
D3	5.1	57%	4.4	52%
D4	3.7	57%	3.8	57%
D5	5	60%	4	52%
D6	2.6	46%	3	45%
D7	2.6	41%	2.7	41%
D8	2.7	44%	2	42%
D9	3.2	32%	2.8	30%
